# Field-driven dynamics and time-resolved measurement of Dzyaloshinskii-Moriya torque in canted antiferromagnet YFeO_3_

**DOI:** 10.1038/s41598-017-04883-3

**Published:** 2017-07-03

**Authors:** Tae Heon Kim, Peter Grüenberg, S. H. Han, B. K. Cho

**Affiliations:** 1 School of Materials Science and Engineering, Gwangju Institute of Science and Technology (GIST), Gwangju, 500-712 Republic of Korea; 2 Gruenberg Center for Magnetic Nanomaterials, Gwangju Institute of Science and Technology (GIST), Gwangju, 500-712 Republic of Korea; 3 Division of Navigation Science, Mokpo National Maritime University, Mokpo, 58628 Republic of Korea

## Abstract

Electrical spin switching in an antiferromagnet is one of the key issues for both academic interest and industrial demand in new-type spin devices because an antiferromagnetic system has a negligible stray field due to an alternating sign between sub-lattices, in contrast to a ferromagnetic system. Naturally, questions arise regarding how fast and, simultaneously, how robustly the magnetization can be switched by external stimuli, e.g., magnetic field and spin current. First, the exploitation of ultrafast precessional motion of magnetization in antiferromagnetic oxide has been studied intensively. Regarding robustness, the so-called inertia-driven switching scenario has been generally accepted as the switching mechanism in antiferromagnet system. However, in order to understand the switching dynamics in a canted antiferromagnet, excited by magnetic field, accurate equation of motion and corresponding interpretation are necessary. Here, we re-investigate the inertia-driven switching process, triggered by the strict phase matching between effective driving field, d***h***/dt, and antiferromagnetic order parameters, ***l***. Such theoretical approaches make it possible to observe the static parameters of an antiferromagnet, hosting Dzyaloshinskii–Moriya (DM) interaction. Indeed, we estimate successfully static parameters, such as DM, exchange, and anisotropy energies, from dynamical behaviour in YFeO_3_, studied using terahertz time-domain spectroscopy.

## Introduction

Because the precessional motion of magnetization has been generally employed for fast magnetization switching^1–5^, much attention has been paid to antiferromagnetic oxide system because of its ultrafast spin response (${\omega }_{{\rm{AF}}}\sim \sqrt{{JK}}\sim {10}^{12}$ s^−1^), coupled with large exchange energy, *J*, and anisotropy energy, *K* ^6^; such characteristics highlight its potential applicability^7–20^. The exchange interaction is found not to contribute to the precession in ferromagnetic system (${\omega }_{{\rm{F}}}\sim \sqrt{{K}}\sim {10}^{9}$ s^−1^)^14, 21^.

Moreover, inertia-driven switching in an antiferromagnet is suggested as a new switching scenario^10, 13, 22^; even after external magnetic field has been turned off, accumulated exchange energy by small disturbances works as a driving force to switch magnetization. More quantitatively, S. Wienholdt *et al*. have constructed the energetic consideration for switching; switching occurs always when the exchange gain (or kinetic energy) stored by the magnetic field is over the anisotropic (or potential) barrier^13^.

Supporting inertia-like behaviour, there are several reports for the spin-current-driven switching in simple antiferromagnet^16^ and canted antiferromagnet^19^ with broken inversion symmetry^23^. Their works highlight the potential for practical applications by replacing the magnetic field with spin current.

However, we reconsider field-driven dynamics in canted antiferromagnet; magnetic resonances are known to exist in two branches^24^ and to be selectively excited by the polarization of external stimulus: magnetic field or spin current. As a result, we found that inertia-driven switching is not induced by a magnetic field ***h***(t) when magnetic field is applied, so that a reliable equation of motion for canted antiferromagnets is necessary to be set up.

Here, we investigated the field-driven dynamics in canted antiferromagnets in two regimes: a field-interaction regime and free-induction decay regime. It is found that the magnetization switching is achieved under the strict phase matching between antiferromagnetic order parameters, ***l*** **=**(***s***_1_-***s***_2_)/2 and driving field, i.e., ~d***h***(t)/dt, consistent to the fact that antiferromagnet dynamics are fundamentally inertia-driven. The ferromagnetic order parameter, ***m*** **=**(***s***_1_ + ***s***_2_)/2 is only a slave vector. In free-induction decay regime, we demonstrate in both experiment and theory that the precessional ellipticity in Sigma mode (S-mode), one of two resonant modes^24^, provides Dzyaloshinskii-Moriya (DM) energy information. The energy information is important because probing DM energy has massive potential for applications, based on the chiral spin domain^25–27^ and antiferromagnetic bubble dynamics^28, 29^ beyond the sub-lattice structure of an antiferromagnet.

## Theory

### Field-driven spin dynamics of YFeO_3_

In this article, we study single crystal YFeO_3_, a prototype for canted antiferromagnet. The magnetism of YFeO_3_ is governed by the Fe^3+^ spins. Assuming that the spatial gradient of magnetization is absent, the magnetic properties could be described as the total energy, *U*, consisting of two sub-lattices, *i* = 1 and 2:

1 $$\begin{array}{rcl}U & = & J{{\boldsymbol{s}}}_{i}\cdot {{\boldsymbol{s}}}_{3-i}+D\cdot ({{\boldsymbol{s}}}_{i}\times {{\boldsymbol{s}}}_{3-i})+{K}_{x}[{({s}_{i,x})}^{2}+{({s}_{3-i,x})}^{2}]\\  &  & +{K}_{z}[{({s}_{i,z})}^{2}+{({s}_{3-i,z})}^{2}]+g{u}_{B}({{\boldsymbol{s}}}_{i}+{{\boldsymbol{s}}}_{3-i})\cdot {\boldsymbol{h}}.\end{array}$$

**Figure 1:**
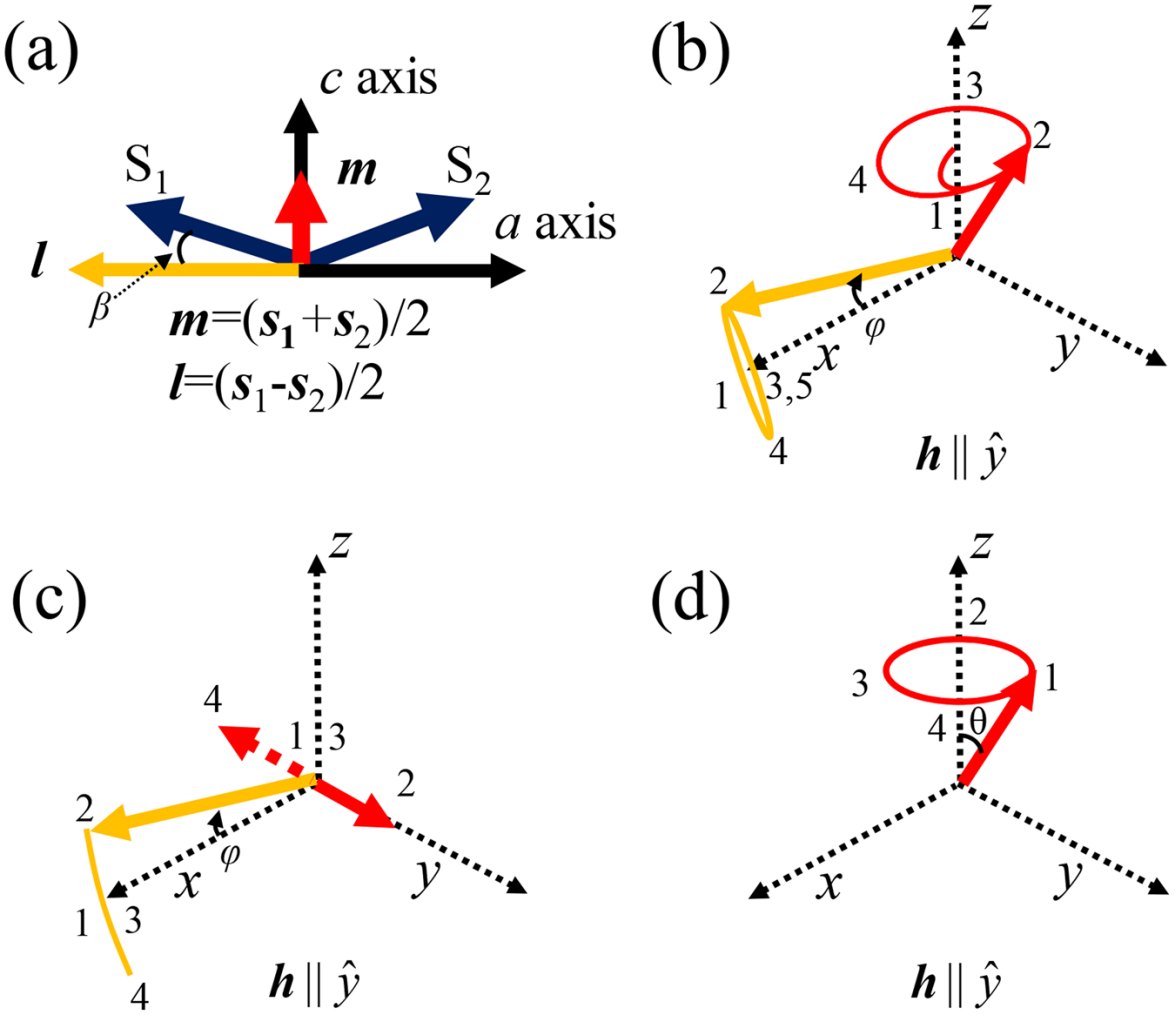
Spin configuration of a canted antiferromagnet in equilibrium and schematic for the excitation modes in various magnetic systems. (**a**) Equilibrium state of a canted antiferromagnet. (**b–d**) Excitation modes when the incident magnetic pulse is applied in the *y*-direction: (**b**) Elliptical precession in a canted antiferromagnet. (**c**) Fluctuating motion in a simple antiferromagnet and (**d**) ferromagnet precession.

The sub-lattices are normalized by their magnitude, e.g., ***s***_**i**_ = ***S***_**i**_/|***S***_**i**_|. The first term denotes exchange energy, where the nearest-neighbour exchange constant, *J*, has 63.7 meV. The second term describes DM energy, where the DM vector, ***D***, is $-{{D}}_{{\rm{y}}}\hat{y}$ with *D*_y_ = 1.4 meV. The third and fourth terms are two anisotropy energies where *K*_x_ and *K*_z_ are set to be 22 *μ*eV and 9.9 *μ*eV respectively. These energy combinations give rise to weak ferromagnetism where the anti-parallel spins are tilted slightly towards the *z*-axis in Fig. 1(a). The final term is Zeeman energy, where *g* is Landė *g*-factor, and *u*_B_ is Bohr magneton, which is equal to the multiplication of gyromagnetic ratio, *γ*, and reduced Plank constant, *ħ*. The dynamics for our magnetic system can be described by coupled Landau-Lifshitz-Gilbert (LLG) equation:

2 $${\dot{{\boldsymbol{s}}}}_{{\rm{i}}}=[{J}{{\boldsymbol{s}}}_{{\rm{i}}}\times {{\boldsymbol{s}}}_{3-{\rm{i}}}+{(-1)}^{{\rm{i}}+1}{{\boldsymbol{s}}}_{{\rm{i}}}\cdot ({\boldsymbol{D}}\times {{\boldsymbol{s}}}_{3-{\rm{i}}})+{{K}}_{{\rm{x}}}\hat{x}\times {{\boldsymbol{s}}}_{i}+{{K}}_{{\rm{z}}}\hat{z}\times {{\boldsymbol{s}}}_{{\rm{i}}}]/\hslash +\gamma ({{\boldsymbol{s}}}_{{\rm{i}}}+{{\boldsymbol{s}}}_{3-{\rm{i}}})\times {\boldsymbol{h}}+\alpha ({{\boldsymbol{s}}}_{i}\times {\dot{{\boldsymbol{s}}}}_{i}),$$

where the final term is magnetic damping characterized by damping coefficient, *α*.

Next, with ***l*** = (***s***_1_ − ***s***_2_)/2 and ***m*** = (***s***_1_ + ***s***_2_)/2, equation (2) can be written as:

3 $$\begin{array}{rcl}\dot{{\boldsymbol{m}}} & = & [-{\boldsymbol{D}}\times ({\boldsymbol{m}}\times {\boldsymbol{l}})+{{K}}_{{\rm{x}}}\hat{x}\times ({m}_{{\rm{x}}}{\boldsymbol{m}}+{l}_{{\rm{x}}}{\boldsymbol{l}})+{{K}}_{{\rm{z}}}\hat{z}\times ({m}_{{\rm{z}}}{\boldsymbol{m}}+{l}_{{\rm{z}}}{\boldsymbol{l}})]/\hslash \\  &  & +\gamma ({\boldsymbol{m}}\times {\boldsymbol{h}})+\alpha ({\boldsymbol{l}}\times \dot{{\boldsymbol{l}}}+{\boldsymbol{m}}\times \dot{{\boldsymbol{m}}}),\end{array}$$

4 $$\begin{array}{rcl}\dot{{\boldsymbol{l}}} & = & [2{J}({\boldsymbol{l}}\times {\boldsymbol{m}})+{\boldsymbol{l}}\times ({\boldsymbol{l}}\times {\boldsymbol{D}})-{\boldsymbol{m}}\times ({\boldsymbol{m}}\times {\boldsymbol{D}})+{{K}}_{{\rm{x}}}\hat{x}\times ({m}_{{\rm{x}}}{\boldsymbol{l}}+{l}_{{\rm{x}}}{\boldsymbol{m}})\\  &  & +{{K}}_{z}\hat{z}\times ({m}_{z}{\boldsymbol{l}}+{l}_{z}{\boldsymbol{m}})]/\hslash +\gamma ({\boldsymbol{l}}\times {\boldsymbol{h}})+\alpha ({\boldsymbol{m}}\times \dot{{\boldsymbol{l}}}+{\boldsymbol{l}}\times \dot{{\boldsymbol{m}}}).\end{array}$$

In canted antiferromagnets, two resonant modes, named as S-mode and Gamma-mode (G-mode)^24^, are excited selectively depending on external magnetic field polarization parallel or perpendicular to the *z*-axis. Here, we consider the S-mode when magnetic field is applied along the *y*-axis. With the effective variables, {*l*_x_, *m*_y_, *l*_z_}, the following approximations can be exploited: ***m*** · ***l*** = 0, |***m***|^2^ + |***l***|^2^ = 1, |***m***| ≪ |***l***| and ${l}^{2}\sim 1\to {\boldsymbol{l}}\cdot \dot{{\boldsymbol{l}}}\sim 0$ ^30–34^. In addition, these terms coupled with anisotropy energies can be ignored because |*K*_x_| and |*K*_z_| ≪ *D*_y_ < *J*. Taking cross product of ***l*** in equation (4), we obtain the analytical relations between ***m*** and ***l***:

5 $$\frac{\dot{{\boldsymbol{l}}}\times {\boldsymbol{l}}}{2{J}/\hslash }\sim (\frac{-{{D}}_{y}{l}_{z}}{2{J}},\frac{-{l}_{z}{\dot{l}}_{x}+{l}_{x}{\dot{l}}_{z}}{2{J}/\hslash }+\frac{g{u}_{{\rm{B}}}{h}_{y}}{2{J}},\frac{{{D}}_{y}{l}_{x}}{2{J}})={\boldsymbol{m}}$$

Because *m*_x_ (or *m*_z_) is only coupled with *l*_z_ (or *l*_x_), we anticipate ***l***’s dynamics through the slave vector, ***m***. The dynamic equation of motion in G-mode is described in detail in the Supplementary information.

In S-mode, ***m*** appears to precess along the *y*-axis in a manner similar to ferromagnet precession in Fig. 1(d). However, the precession of ***m*** is combined with fluctuating motion with two different origins. For example, *m*_y_ is caused by the precession of excited simple antiferromagnet, as in Fig. 1(c). That is, when sub-lattice spins are precessing symmetrically along anisotropic field directions, the magnetic component parallel to the magnetic field direction is in-phase and reinforced, but the other is out-of-phase and cancelled out. However, *m*_x_ and *m*_z_ are induced by asymmetric motion of spins because of DM torque. Therefore, the two-dimensional trajectory, *m*_xy_, is inherently elliptical.

Substituting *m*_y_ in equation (5) into equation (3), we have the 2D pendulum equation on ***l*** = (*l*_*x*_, *l*_*z*_) = (cos[*φ*], sin[*φ*]):

6 $$\ddot{\phi }+\dot{\phi }2\alpha {J}/\hslash +\,\sin \,[2\phi ]{\omega }_{{\rm{Sigma}}}^{2}/2=\gamma {\dot{h}}_{y},$$

where ${\omega }_{{\rm{Sigma}}}^{2}=2J({{K}}_{{\rm{x}}}-{{K}}_{{\rm{z}}})/{\hslash }^{2}$. Equation (6) is identical to the equation of motion in simple antiferromagnets because same effective variables are used. In S-mode, the role of DM interaction is to create *m*_z_ (~*l*_x_) and *m*_x_(~*l*_z_) components, whereas in G-mode, DM interaction lifts the degeneracy of simple antiferromagnets (see the Supplementary information). Although LLG equations are the first-order differential equation of motion with respect to time, the equation of motion for antiferromagnets is of second order because *J* > 0. Therefore, we could expect inertia-like motion.

## Results and Discussion

### Field-interaction regime

**Figure 2:**
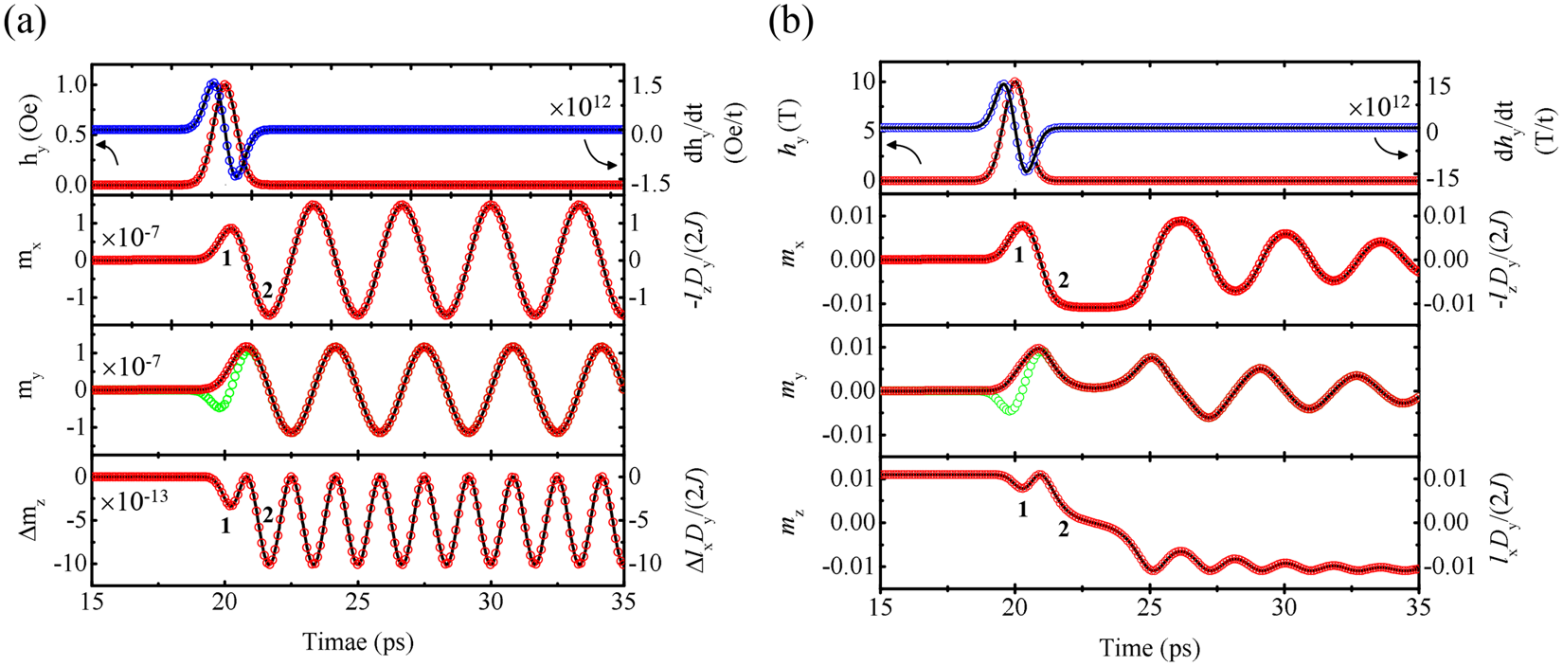
Analytical and numerical calculation results. (**a**) Excitation modes and (**b**) switching modes via a Gaussian-type magnetic pulse in a canted antiferromagnet. The solid line and open circles represent the numerical and analytical solution, respectively. Note that two tipping processes, denoted by 1 and 2 in *m*_z_ and *m*_x_, are ascribed to the resonant effect by a single-cycle differential field, d*h*_y_/dt (blue), not by *h*_y_ (red). Magnetization switching occurs by process 2. *m*_y_ also shows two tipping processes (green) when the field-induced magnetization, Δ*m*_y_ = *gu*_B_ *h*_y_/(2*J*), is excluded.

Both models are numerically calculated with the time interval of Δ*t* = 0.01 ps and in a time window of 15 ps ≤ *t* ≤ 35 ps. A Gaussian-type magnetic pulse, *h*_y_(t), in the form of ${h}_{{\rm{y}}}(t)={H}_{0}\exp [-\frac{{(t-{t}_{0})}^{2}}{2{\sigma }_{{\rm{t}}}^{2}}]$, is applied for a center of peak with *t*_0_ = 20 ps and temporal pulse width, *σ*_t_ = 1 ps. We choose the parameters for peak amplitude, *H*_0_ and *α*, to be [*H*_0_, *α*] = [1 Oe, 0] for excitation mode and [1 T, 0.001] for switching mode, respectively. Here, damping constants, *α* = 0, 0.001 are arbitrarily chosen to focus on the interplay between d***h***/dt and magnetization although estimated damping constant for YFeO_3_ is 0.0003. The parameters, ***l*** and ***m*** in the pendulum model (open circle), are produced from the resultant *φ*(*t*) with a relation to equation (5) and are found to be identical to those in LLG model (solid line). The equation of motion of pendulum confirms that the differential field, d*h*_y_(t)/dt, functions as driving torque where the differential form of Gaussian pulse is of single-cycle shape. As a result, *m*_z_ and *m*_x_ (or *l*_x_ and *l*_z_) are tipped twice, as denoted by 1 and 2 (see Fig. 2(a) and (b)). These consecutive tips occur resonantly via the single-cycle torque of d*h*_y_(t)/dt.

Note that *m*_y_ is coupled with *l*_x_ and *l*_z_, together with the field-induced magnetization, ${\rm{\Delta }}{m}_{{\rm{y}}}=g{u}_{{\rm{B}}}{h}_{{\rm{y}}}/(2{J})$ in equation (6). When Δ*m*_y_ is removed, we can easily see the consecutive tips in *m*_y_, as shown in Fig. 2 (open green circles). Experimentally, Δ*m*_y_ would be included in a transient Faraday rotation signal (linearly proportional to ***m***) as a strong transient in the canted antiferromagnet^35AQ1–38^, or simple antiferromagnet^39^. Assuming that the other optical effects associated with the strong transients are completely excluded, the exact phase or the maximum amplitude of ***l*** should be observed in Faraday rotation signal without Δ*m*_y_.

**Figure 3:**
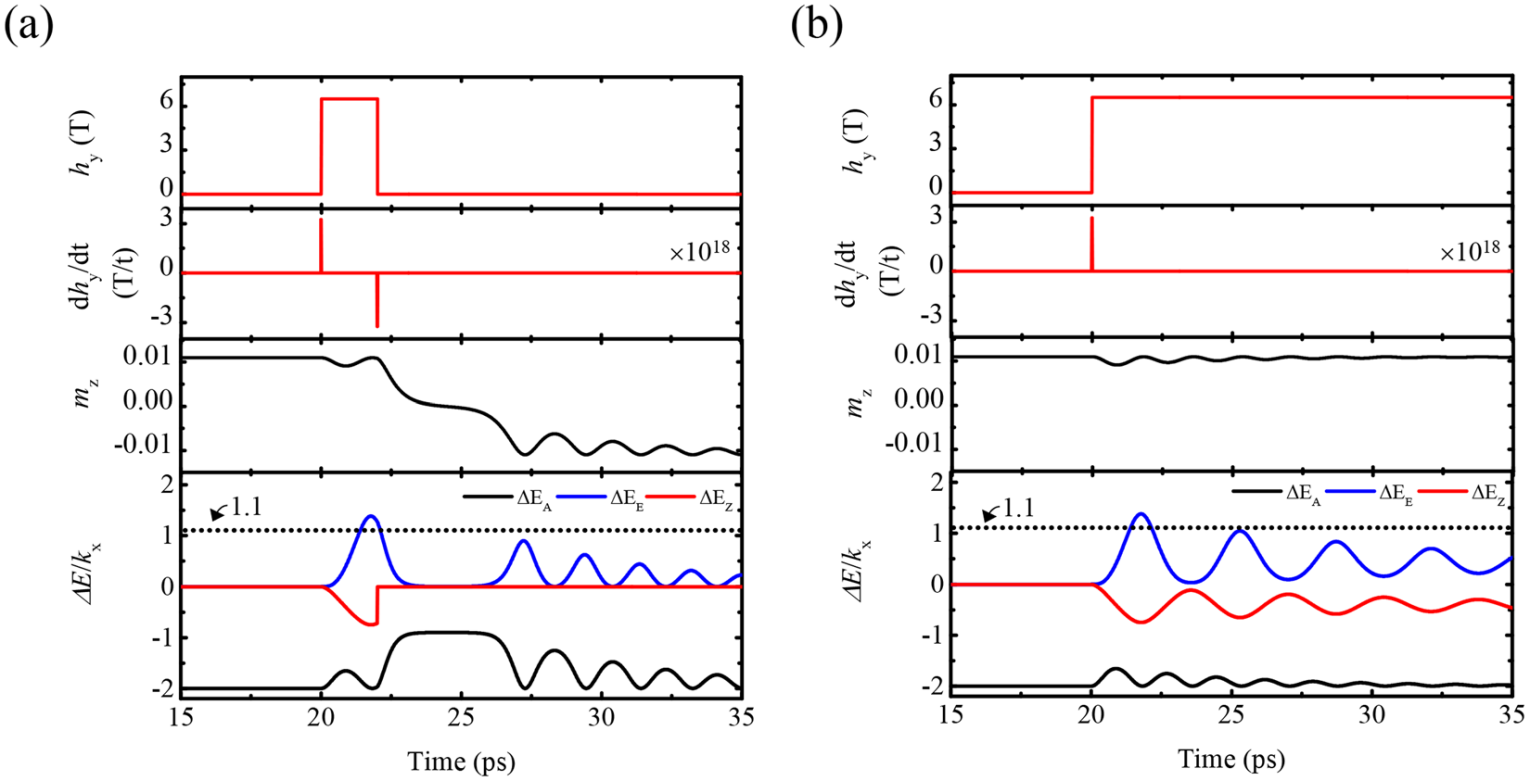
Energetic analysis for switching mode and non-switching mode using two different magnetic fields in canted antiferromagnets. The differential fields, d*h*_y_/dt in two dynamic modes are of single-cycle (**a**) and half-cycle (**b**) forms. Three energy differences (exchange, anisotropy and Zeeman energies) are plotted as Δ*E*_E_ = 2*J*[***s***_1_(t) · ***s***_2_(t) − ***s***_1_(0) · ***s***_2_(0)], Δ*E*_A_ = −{*K*_x_[*s*_1,*x*_(t)^2^ + *s*_2,*x*_(t)^2^] − *K*_z_[*s*_1,*z*_(t)^2^ + *s*_2,*z*_(t)^2^]} and ${\rm{\Delta }}{E}_{{\rm{Z}}}=g{u}_{B}[{{\boldsymbol{s}}}_{1}({\rm{t}})+{{\boldsymbol{s}}}_{2}({\rm{t}})]\cdot {h}_{{\rm{y}}}\hat{y}$ respectively.

To examine the switching process, we analyse canted antiferromagnet dynamics energetically. Two static magnetic fields of *H* = 6.5 T are turned on along the *y*-axis at *t* = 20 ps and one of them is turned off after Δ*t* = 2 ps. Several energy differences are defined and plotted in the fourth row of Fig. 3(a,b): exchange gain, ${\rm{\Delta }}{E}_{{\rm{E}}}=2{J}[{{\boldsymbol{s}}}_{1}({\rm{t}})\cdot {{\boldsymbol{s}}}_{2}({\rm{t}})-{{\boldsymbol{s}}}_{1}(0)\cdot {{\boldsymbol{s}}}_{2}(0)]$, anisotropy barrier, ${\rm{\Delta }}{E}_{{\rm{A}}}=-\{{{K}}_{{\rm{x}}}[{s}_{1,x}{({\rm{t}})}^{2}+{s}_{2,x}{({\rm{t}})}^{2}]-{{K}}_{{\rm{z}}}[{s}_{1,z}{({\rm{t}})}^{2}\,+$ ${s}_{2,z}{({\rm{t}})}^{2}]\}$, and Zeeman energy, ${\rm{\Delta }}{E}_{{\rm{Z}}}=g{u}_{B}[{{\boldsymbol{s}}}_{1}({\rm{t}})+{{\boldsymbol{s}}}_{2}({\rm{t}})]\cdot {h}_{{\rm{y}}}\hat{y}$. So far, it is known that the inertia-driven switching occurs once Δ*E*_E_, accumulated from a decrease of Δ*E*_Z_, overcomes potential barrier^13^. For a system with two anisotropies as like YFeO_3_, the potential barrier is estimated as |2(*K*_x_ − *K*_z_)/*K*_x_| = 1.1 (see the fourth low of Fig. 3(a,b)). Although both excitations show identical behaviour until *t* = 22 ps, the trajectory of *m*_z_ confirms that d***h***/dt|_t=22ps_ (see Fig. 3(a)) contributes to magnetization switching. As long as the field is turned on, any torque does not occur because |d*h*/dt| = 0. Therefore, the strict phase matching between ***l*** and $\gamma \dot{{\boldsymbol{h}}}$ (or ~***p***(t) of Slonczewski-type spin transfer torque^16, 34^) plays a main role in the switching process.

### Free induction decay regime

Next, we focus on the spin dynamics, which is driven only by an internal field (*h*_y_ = 0). In particular, the precessional trajectory of S-mode provides the information of DM energy as described in equation (5). With the consideration of experimental condition, where DC magnetic field of *h*_z,DC_ ~ −97.5 Oe is applied along the *z*-axis for magnetization saturation, equation (6) is changed as

7 $${\boldsymbol{m}} \sim (\frac{-{D}_{{\rm{y}}}{l}_{{\rm{z}}}-g{u}_{{\rm{B}}}{h}_{{\rm{z}},{\rm{DC}}}{l}_{{\rm{z}}}{l}_{{\rm{x}}}}{2J},\frac{-{l}_{{\rm{z}}}{\dot{l}}_{{\rm{x}}}+{l}_{{\rm{x}}}{\dot{l}}_{{\rm{z}}}}{2J/\hslash },\frac{{D}_{{\rm{y}}}{l}_{{\rm{x}}}+g{u}_{{\rm{B}}}{h}_{{\rm{z}},{\rm{DC}}}{l}_{{\rm{x}}}^{2}}{2J})$$

However, the magnetization dynamics are driven effectively by internal field because of ${D}_{{\rm{y}}}\gg g{u}_{{\rm{B}}}{h}_{{\rm{z}},{\rm{DC}}}$ and, still, ***l*** ≫ ***m***. In addition, equation (6) is not changed by a weak DC-magnetic field because d*h*_z,DC_/dt = 0. Therefore, the ellipticity, $\varepsilon \equiv |{m}_{{\rm{y}}}|/|{m}_{{\rm{x}}}|$, of the precessional motion is deduced as $[-2J/({D}_{y}{l}_{z})][\hslash (-{l}_{{\rm{z}}}{\dot{l}}_{{\rm{x}}}+{l}_{{\rm{x}}}{\dot{l}}_{{\rm{z}}})/(2J)]$ $\sim C\hslash {\omega }_{{\rm{Sigma}}}\,\cos \,[{\omega }_{{\rm{Sigma}}}t]/({D}_{{\rm{y}}}\,\sin \,[C\,\sin \,[{\omega }_{{\rm{Sigma}}}t]])$ and, thereby, $\sim \hslash {\omega }_{{\rm{Sigma}}}/({D}_{{\rm{y}}}\,\tan \,[{\omega }_{{\rm{Sigma}}}t])$ when the S-mode is weakly excited or $\phi  \sim C\,\sin \,[{\omega }_{{\rm{Sigma}}}t]$ is small.

**Figure 4:**
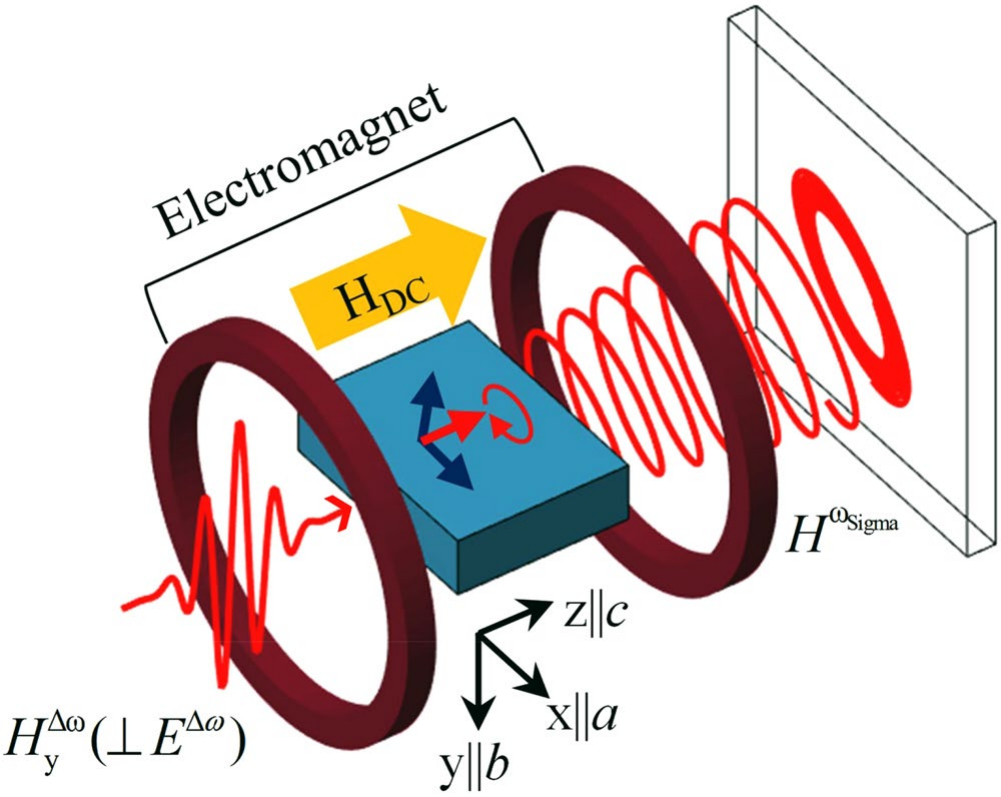
Schematic diagram for THz emission in YFeO_3_ after excitation by a vertically polarized magnetic pulse of light, ${{H}}_{{\rm{y}}}^{{\rm{\Delta }}{\rm{\omega }}}$. When ${{H}}_{{\rm{y}}}^{{\rm{\Delta }}{\rm{\omega }}}$ is incident on the sample, the magnetizations are tilted away from their equilibrium position; subsequently, they return to the original position, precessing at a frequency of 0.3 THz via the internal magnetic field, and emitting a free-induction decay signal as the elliptically polarized light. Here, the DC magnetic field, *h*_z,DC_, from the Helmholtz-type coils is used for magnetization saturation in the direction of the -*z*-axis or the crystalline *c*-axis. We set the crystalline axes, *a*, *b*, and *c* to correspond to the Cartesian axes, *x*, *y*, and *z*.

Our analytical approaches to YFeO_3_ are appropriate because *D*_y_/*J* ratio is weak enough or canting angle, *β*, is small where $\beta =\arctan [{m}_{{\rm{x}}}/{l}_{{\rm{x}}}]=\arctan [{D}_{{\rm{y}}}/(2J)] \sim 0.6$ degrees. As the *D*_y_/*J* ratio increases, the pendulum model deviates gradually from LLG model. At room temperature, the antiferromagnetic spin state of YFeO_3_ is illustrated in Fig. 1(a). YFeO_3_ exhibits two resonant modes, S-mode and G-mode^24^. Terahertz (THz) time-domain spectroscopy is used to measure the DM energy in S-mode, where we set the crystalline axes, *a*, *b*, and *c* corresponding to Cartesian axes, *x*, *y*, and *z* (see Fig. 4). When vertically polarized THz magnetic pulse (*h*_y_//*b*) transmits through YFeO_3_ with a thickness of 1.5 mm^40^, ***l*** experiences a driving toque and is tilted effectively by the spectral component around resonant frequency. Simultaneously, ***m*** starts to precess and oscillate along the *z*-axis due to internal magnetic fields. The oscillating frequency is higher by two times than the precessional frequency, while the oscillating amplitude is negligible in weak excitation^19^. Precession motion will stop eventually because of damping. This is called as free-induction decay process. Actually, the precession of magnetization emits an electromagnetic or emission wave and a photoconductive antenna detects it.

**Figure 5:**
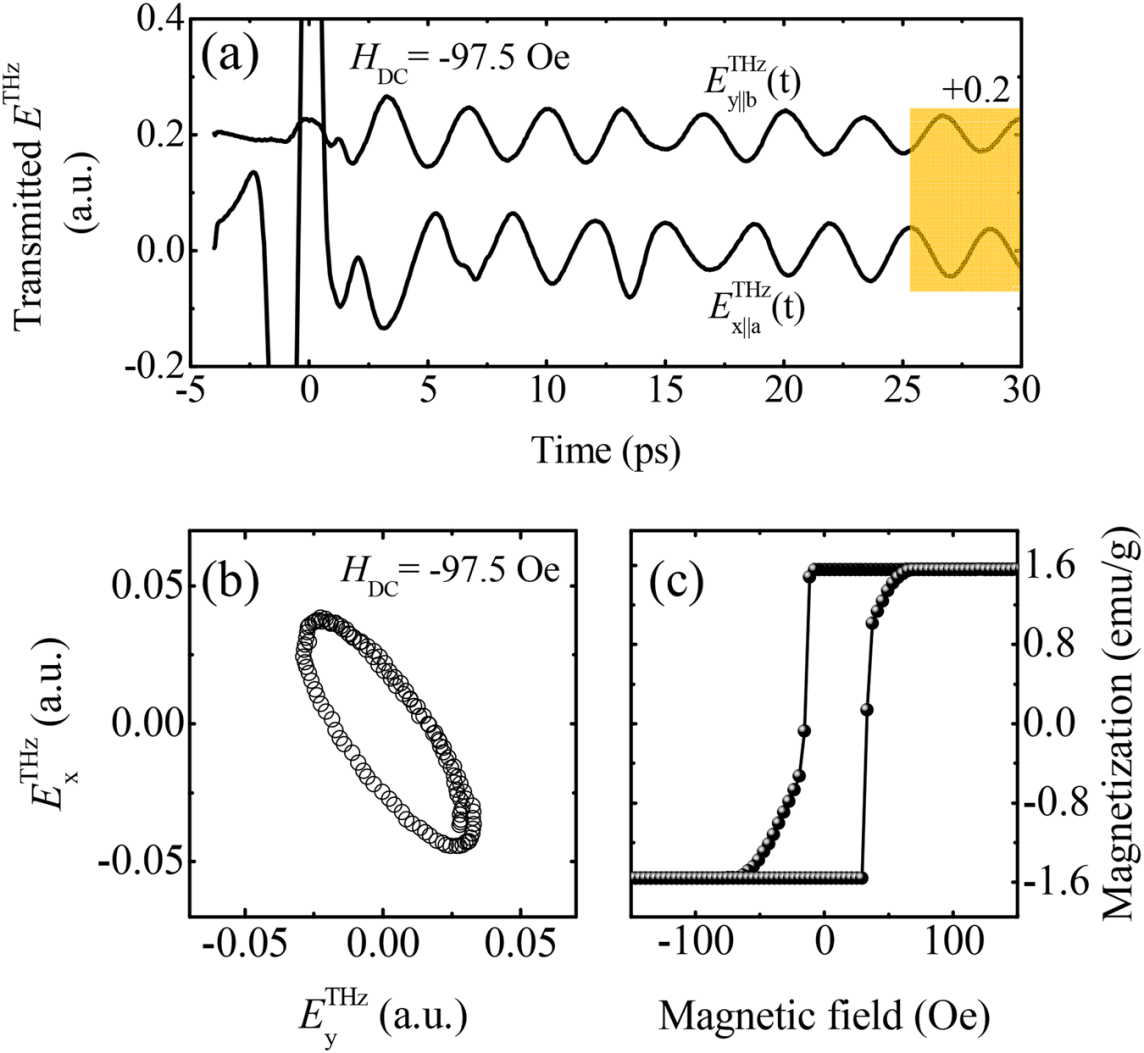
THz emission waves and magnetic hysteresis curve. (**a**) THz waveforms transmitted through the sample when the magnetic field is parallel to the y-axis. YFeO_3_ is saturated fully under the external magnetic field of −97.5 Oe. ${E}_{{\rm{x}}\parallel {\rm{a}}}^{{\rm{THz}}}$ and ${E}_{{\rm{y}}\parallel {\rm{b}}}^{{\rm{THz}}}$ are extracted using a pair of wire-grid polarizers set at 45° and −45° from the *x*-direction. Here, ${E}_{{\rm{y}}\parallel {\rm{b}}}^{{\rm{THz}}}$ is shifted upward by +0.2 for clarity. (**b**) Two-dimensional trajectories of emissions in a temporal window between 25 ps and 30 ps. (**c**) Magnetization hysteresis curve measured using a sample vibrating magnetometer. All data are quoted from ref. 40.

In experiment, free-induction decay signals under *h*_z,DC_ ~ −97.5 Oe for saturation are obtained, as shown in Fig. 5(a), where the raw data are quoted from ref. 40. Here, the incident THz electric field is linearly polarized along the *x*-axis, and detector is only sensitive to the *x*-component of electric field of emission wave. Therefore, a wire grid polarizer is used to extract the *y*-component. After THz field passed through polarizers with the angles of +45 and −45 degree from the *x*-axis, subtraction and summation of the two transmitted THz waves yield the *y*- and *x*-component of emission wave (${E}_{{\rm{x}}}^{{\rm{THz}}}$ and ${E}_{{\rm{y}}}^{{\rm{THz}}}$) in Fig. 5(a), respectively^40, 41^. However, the resultant waves are more strongly elliptical than expected (see Fig. 5(b)). They are modulated by four effects, accumulated during propagation of emission waves through YFeO_3_ crystal. First, the modulation happens due to the refractive index difference, ${\rm{\Delta }}{n}_{{\rm{ab}}}\sim -0.23$, between the *a*-axis and *b*-axis^40, 42^, which results in a phase delay of ${\rm{\Delta }}{n}_{{\rm{ab}}}/c\times t\sim 1.15$ ps between emission waves. Second, the different absorption coefficients or transmissions, *T*, degrade the emission waves by the factors of *T*_a_ ~ 0.41 and *T*_b_ ~ 0.35^42^. Third, refractive index mismatch between incident THz pulse and spin wave, depending on the crystal axis, induces interference after transmission. Therefore, the factors are calculated using the cardinal sine or sinc function: *f*_a_ ~sinc(2*π* × 0.3 THz/*c* × 1.5 mm) ~ 0.99 and *f*_b_ ~ sinc(2*π* × 0.3 × 10^12^ s^−1^/*c* × (−0.21) × 1.5 mm) ~ 0.84, where *c* is the speed of light. Fourth, the spin wave (or emission wave) perpendicular to the incident magnetic field is significantly dependent on the magnetization state^40^. To remove this effect, we saturated magnetization by applying *h*_z,DC_.

**Figure 6:**
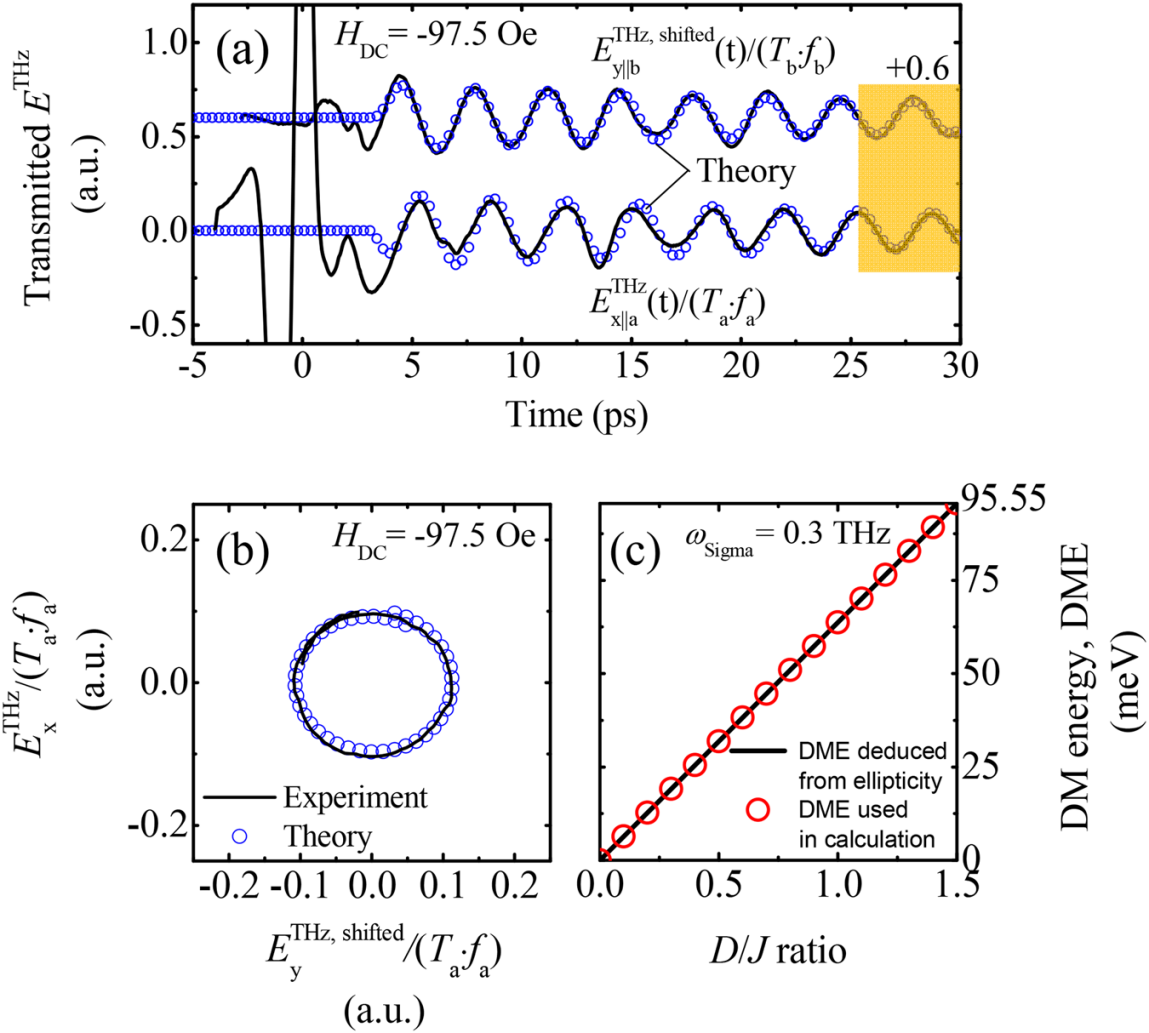
THz emissions scaling linearly with the spin wave trajectories and simulations. (**a**) ${E}_{{\rm{y}}\parallel {\rm{b}}}^{{\rm{THz}},{\rm{shifted}}}$ is the temporal waveform shifted by −1.15 ps from ${E}_{{\rm{y}}\parallel {\rm{b}}}^{{\rm{THz}}}$ because of the refractive index difference, Δn_ab_ = −0.23, between the *a* and *b* axes at 0.3 THz. In addition, the resultant ${E}_{{\rm{x}}\parallel {\rm{a}}}^{{\rm{THz}}}$ and ${E}_{{\rm{y}}\parallel {\rm{b}}}^{{\rm{THz}},{\rm{shifted}}}$ were considered by different transmissions, *T*_a_ = 0.41 and *T*_b_ = 0.35, and phase mismatching factors, *f*_a_ = 0.99 and *f*_b_ = 0.84, which results from the interference between propagating THz pulses and emissions. (**b**) Two-dimensional trajectories of emissions in the temporal window between 25 ps to 30 ps. In (**a**,**b**), the experimental results (solid line) are matched closely to the numerical ones (open circles) calculated by using the parameters extracted from the sample. (**c**) The determination of the various DM energies when the S-mode is weakly excited or a Gaussian-type magnetic pulse with [*H*_0_, *σ*_*t*_, *α*] = [1 Oe, 1 ps, 0] is applied. The DM energy used in the simulation (open circle) is comparable to one deduced from the ellipticity (solid line).

Figure 6(a) shows emission waves, scaled linearly with the real spin wave: ${{E}}_{x||{\rm{a}}}^{{\rm{emission}}}/{{T}}_{{\rm{a}}}/{{f}}_{{\rm{a}}}$ and ${{E}}_{{\rm{y}}||{\rm{b}}}^{{\rm{emission}},{\rm{shifted}}}/{{T}}_{b}/{{f}}_{b}$. From the ellipticity of precessional trajectories in Fig. 6(b), the DM energy is estimated as 0.3 × 10^12^ s^−1^ × 2*π* × *ħ*/*ε* × (1.6 × 10^12^ J/eV)^−1^ ~ 1.4 meV where $\varepsilon \equiv |{m}_{{\rm{y}}}|/|{m}_{{\rm{x}}}|=|{E}_{{\rm{x}}}^{{\rm{THz}}}|/|{E}_{{\rm{y}}}^{{\rm{THz}}}|=0.9178$. Here, the damping effect is ignored because of negligible contribution to the DM energy calculation. (The damping constant is estimated as 0.0003 by fitting the precessional data to LLG model and it is due to the magnon scattering on phonons and spins of Yittrium ions^9^).

The exchange energy, *J*, is deduced using the asymmetric exchange model^40^: *J* = *M*_0_ *D*_y_/*M*_s_ = 72.5 emu/g × 1.4 meV/1.54 emu/g = 63.7 meV, where *M*_0_ is magnetic moment of ions per unit mass, and *M*_s_ is the saturation magnetization in Fig. 5(c). The two anisotropy energies are deduced through the two resonant frequency formulas, where *ω*_Sigma_ = 0.3 THz^15, 40^ and *ω*_Gamma_ = 0.52 THz and found to be ${K}_{{\rm{x}}}=({\omega }_{{\rm{Gamma}}}^{2}{\hslash }^{2}-{D}_{{\rm{y}}}^{2})/(2J) \sim 22\,{\mu }{\rm{eV}}$ and ${K}_{{\rm{z}}}={K}_{{\rm{x}}}-{\omega }_{{\rm{Sigma}}}^{2}{\hslash }^{2}/(2J)\sim 9.9\,{\mu }{\rm{eV}}$. All parameters are in good agreement with reference^43^. Moreover, our numerical calculation using the above parameters explains the experimental data well.

Figure 6(c) shows DM energy, deducted from ellipticity in Fig. 6(b), in terms of *D*/*J* ratio, together with DM energy in calculation. When S-mode is weakly excited or a Gaussian-type magnetic pulse with [*H*_0_, *σ*_*t*_, *α*] = [1 Oe, 1 ps, 0] is applied, the precessional ellipticity, calculated from LLG model, determines exact DM energy. DM energy from ellipticity matches well with that in calculation up to *D*_y_/*J* = 1.5 (or canting angle ~28 degrees), indicating that measurement of the strong DM energy through ellipticity analysis is quite effective experimental method. The value of *D*_y_/*J* = 1.5 makes the method useful to examine the antiferromagnetic bubble and chiral domain wall dynamics and to control the DM energy through interface engineering^44^. Our experimental condition agrees with weak excitation by THz magnetic pulse. The THz electric field strength did not exceed the value of ∼1 kV/cm for a focused beam size of 3 mm; therefore, the peak magnetic field was below 3 Oe. And our magnetic system is directly coupled with the magnetic field. If spin waves are excited by the electric field of THz pulse, the experimental results that Faraday rotation signals in NiO^12^ and emission amplitudes in YFeO_3_^15^ are linearly proportional to the pump field would be conjectured to be linear magneto-electric effect. However, such coupling is not allowed in centrosymmetric system^45^.

## Summary

In this article, we investigate the field-driven dynamics of a canted antiferromagnet in both theory and experiment. In a field-interaction regime, the antiferromagnet dynamics are excited or switched in the strict phase matching condition between ~d***h***/dt and ***l***. In a free-induction decay regime, we found that the precessional ellipticity of S-mode determines DM energy in a canted antiferromagnet system. From experimental ellipticity data, we deduced successfully the DM energy, together with static parameters (*J*, *K*_x_, *K*_z_) in YFeO_3_, using terahertz time-domain spectroscopy. We expect that our results would contribute significantly to broaden our fundamental understanding on antiferromagnet dynamics.

## Electronic supplementary material

Field-driven dynamics and time-resolved measurement of the Dzyaloshinskii-Moriya torque in canted antiferromagnet YFeO3
