# Supplementary material for: Field-driven dynamics and time-resolved measurement of Dzyaloshinskii-Moriya torque in canted antiferromagnet YFeO3
Source: Sci Rep. 2017 Jul 3;7:4515. doi: 10.1038/s41598-017-04883-3 (PMC5495818; doi:10.1038/s41598-017-04883-3)
Supplement: Supplementary file 1 — Field-driven dynamics and time-resolved measurement of the Dzyaloshinskii-Moriya torque in canted antiferromagnet YFeO3 [file 41598_2017_4883_MOESM1_ESM.pdf]

SUPPLEMENTARY INFORMATION

Field-driven dynamics and time-resolved measurement of the Dzyaloshinskii-Moriya torque in canted antiferromagnet $YFeO_3$

Tae Heon Kim[1,2], Peter Grüenberg[2], Song Hee Han[3], and Beong Ki Cho[1,2,a)]

[1]*School of Materials Science and Engineering, Gwangju Institute of Science and Technology (GIST), Gwangju 500-712, Republic of Korea*

[2]*Gruenberg Center for Magnetic Nanomaterials, Gwangju Institute of Science and Technology (GIST), Gwangju 500-712, Republic of Korea*

[3]*Division of Navigation Science, Mokpo National University, Mokpo 58628, Republic of Korea*

[a)] Correspondence and requests for materials should be addressed to B. K. Cho.

(chobk@gist.ac.kr).

Equation of motion in Gamma-mode (G-mode)

Equation of motion for G-mode is derived when the magnetic field is applied in *z*-direction. From the numerical calculation of LLG model, we set effective parameters as like $\boldsymbol{l} = (l_x, l_y, 0)$, $\boldsymbol{m} = (0, 0, m_z)$ and $\boldsymbol{h} = (0, 0, h_z)$. A Gaussian-type magnetic pulse, $h_z(t)$ of the form $h_z(t) = H_0 \exp[-\frac{(t-t_0)^2}{2\sigma_t^2}]$ is applied with the position of the center of the peak, t_0 = 20 ps, temporal pulse width, σ_t = 1 ps, peak amplitude, H_0 = 10 T, and damping constant, $\alpha = 0.001$, respectively. Together with trial solutions, $(l_x, l_y) = (\cos[\varphi], \sin[\varphi])$, taking the cross product of $\boldsymbol{l}$ in equation (4) leads to

$$\frac{\dot{\boldsymbol{l}} \times \boldsymbol{l}}{2J/\hbar} \sim (0, 0, \frac{-l_y \dot{l}_x + l_x \dot{l}_y}{2J/\hbar} + \frac{D_y l_x}{2J} + \frac{g u_B h_z}{2J}) = \boldsymbol{m} \qquad (8)$$

Substituting m_z in equation (8) into equation (3), we also have 2D pendulum equation of motion:

$$\ddot{\varphi} + \dot{\varphi} 2\alpha J / \hbar + \sin[2\varphi]\omega_{\mathrm{Gamma}}^2 / 2 = -\gamma h_{\mathrm{z}} \cos[\varphi] D_{\mathrm{y}} / \hbar + \gamma \dot{h}_{\mathrm{z}}, \qquad (9)$$

where $\omega_{\mathrm{Gamma}}{}^2 = 2J(K_x + D_y{}^2 / 2J) / \hbar^2$.

In G-mode, m_{z} is coupled with l_{x} and l_{y}, together with the field-induced magnetization, $\Delta m_{\mathrm{z}} = g u_{\mathrm{B}} h_{\mathrm{z}} / (2J)$ in equation (8). When Δm_{z} is removed, the consecutive tips in m_{z} are shown as denoted by 1 and 2 (see Fig. S1, open green circle). Although $m_{\mathbf{z}}$ appears to oscillate along the *z*-axis, the trajectory of m_{z} is combined with two spectral components, which are caused by different origins. First, the symmetric precession of spins by anisotropic field induces oscillation with a frequency of ω_{Gamma}. Second, DM field, $(-1)^{\mathrm{i}} \boldsymbol{D} \times \boldsymbol{s}_{\mathrm{i+1}}$ (i = 1 or 2) breaks symmetric motions of spins and thereby induces the second harmonic oscillation, $2\omega_{\mathrm{Gamma}}$, which is ascribed to $D_{\mathrm{y}} l_{\mathrm{x}} / (2J)$, as shown in the third row of Fig. S1.

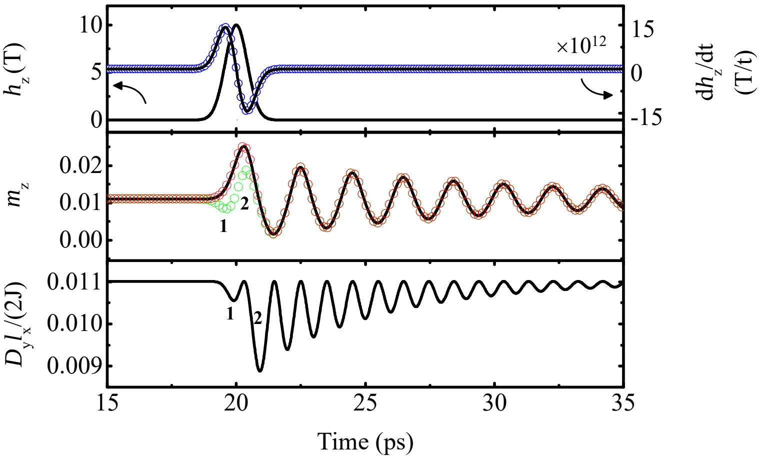

FIG. S1. Analytical and numerical calculation results in G-mode. The solid line and open circles represent the numerical and analytical solution, respectively. m_{z} shows two tipping processes (green) when the field-induced magnetization, $\Delta m_{\mathrm{z}} = g u_{\mathrm{B}} h_{\mathrm{z}} / (2J)$, is excluded.
